# Outcomes in patients with atypical hemolytic uremic syndrome treated with eculizumab in a long-term observational study

**DOI:** 10.1186/s12882-019-1314-1

**Published:** 2019-04-10

**Authors:** Jan Menne, Yahsou Delmas, Fadi Fakhouri, Christoph Licht, Åsa Lommelé, Enrico E. Minetti, François Provôt, Eric Rondeau, Neil S. Sheerin, Jimmy Wang, Laurent E. Weekers, Larry A. Greenbaum

**Affiliations:** 1 Department of Nephrology and Hypertension, Klinik für Nieren- und Hochdruckerkrankungen, Carl Neuberg Str. 1, 30625 Hannover, Germany; 2 Service de Néphrologie Transplantation Dialyse, CHU de Bordeaux, Place Amélie Raba Léon, CEDEX 33076 Bordeaux, France; 3 Department of Nephrology and Immunology, UMR 643, CHU de Nantes, 27 Rue la Pérouse, CEDEX 1 44000 Nantes, France; 4 Division of Nephrology, The Hospital for Sick Children, 555 University Avenue, Toronto, Ontario M5G 1X8 Canada; 5 Alexion Pharma GmbH, Giesshübelstrasse 30, 08045 Zurich, Switzerland; 6 Department of Nephrology, Niguarda Hospital, Piazza Ospedale Maggiore 3, 20162 Milan, Italy; 7 Department of Nephrology, CHU de Lille, 2 Avenue Oscar Lambret, 59000 Lille, France; 8 Urgences Néphrologiques et Transplantation Rénale, Hôpital Tenon, AP-HP, 4 Rue de la Chine, 75020 Paris, France; 9 Sorbonne Université, 15-21 Rue de l’École de Médecine, Paris, 75006 France; 10 Institute of Cellular Medicine, University of Newcastle upon Tyne, 4th Floor, William Leech Building, Newcastle upon Tyne, NE2 4HH UK; 11 Alexion Pharmaceuticals, Inc., 121 Seaport Boulevard, Boston, MA 02210 USA; 12 Néphrologie-Transplantation, CHU de Liège, Sart-Tilman B35, 04000 Liège, Belgium; 13 Division of Pediatric Nephrology, Emory University School of Medicine and Children’s Healthcare of Atlanta, 2015 Uppergate Drive NE, Atlanta, GA 30322 USA

**Keywords:** Atypical hemolytic uremic syndrome, Alternate complement pathway, Eculizumab, Thrombotic microangiopathy

## Abstract

**Background:**

There are limited long-term outcome data in eculizumab-treated patients with atypical hemolytic uremic syndrome (aHUS). We report final results from the largest prospective, observational, multicenter study of patients with aHUS treated with eculizumab.

**Methods:**

Patients with aHUS who participated in any of five parent eculizumab trials and received at least one eculizumab infusion were eligible for enrollment in a long-term follow-up study. Rates of thrombotic microangiopathy (TMA) manifestations off versus on eculizumab were evaluated. Additional endpoints included change from baseline estimated glomerular filtration rate (eGFR), long-term renal outcomes, and serious targeted treatment-emergent adverse events.

**Results:**

Among 93 patients (0–80 years of age), 51 (55%) remained on eculizumab and 42 (45%) discontinued; for those who discontinued, 21 (50%) reinitiated therapy. Patients who reinitiated eculizumab had similar baseline clinical characteristics to patients who remained on eculizumab, with higher likelihood of genetic/autoimmune complement abnormalities, more prior TMAs, and longer disease course versus those who did not reinitiate. Mean eGFR improved rapidly and remained stable for up to 6 years on eculizumab. In patients who discontinued, there was a trend toward decreasing renal function over time from discontinuation. Additionally, off-treatment TMA manifestation rates were higher in those aged < 18 years at diagnosis, with identified genetic/autoimmune complement abnormalities, or history of multiple TMAs prior to eculizumab initiation. The safety profile was consistent with previous studies. Three definite and one possible meningococcal infections related to eculizumab were reported and resolved with treatment. Three deaths unrelated to eculizumab were reported.

**Conclusions:**

The current study confirms the efficacy and safety of eculizumab in aHUS, particularly with regard to long-term renal function and TMA events. Pediatric age at disease onset and presence of genetic or autoimmune complement abnormalities are risk factors for TMA events off treatment. Overall, patients who discontinue eculizumab may be at risk for additional TMA manifestations and renal function decreases. Discontinuation of eculizumab, with careful monitoring, is an option in select patients with consideration of patient preference, organ function normalization, and risk factors for relapse, including mutational analysis, age of onset, and history of multiple TMA episodes.

**Trial registration:**

ClinicalTrials.gov NCT01522170, January 31, 2012.

**Electronic supplementary material:**

The online version of this article (10.1186/s12882-019-1314-1) contains supplementary material, which is available to authorized users.

## Background

Atypical hemolytic uremic syndrome (aHUS) is a rare disorder caused by overactivation of the alternative pathway of complement and is primarily characterized by thrombotic microangiopathy (TMA) [1, 2]. Classic manifestations include thrombocytopenia, microangiopathic hemolytic anemia, and acute kidney injury, although other organs are often also affected. During the era when plasma infusion or plasma exchange was the mainstay of management, aHUS was associated with a poor prognosis. Historically, 36% of children and 64% of adults developed end-stage renal disease or died within 3 to 5 years of disease onset [3].

Eculizumab (Soliris®, Alexion Pharmaceuticals, Inc., Boston, MA, USA) is a humanized monoclonal complement inhibitor that is the first and only approved treatment for patients with aHUS [4, 5]. Eculizumab binds with high affinity to C5, inhibiting C5 cleavage to C5a and C5b and preventing the generation of the terminal complement complex C5b-9, thus inhibiting complement-mediated TMA. Eculizumab was proven to be effective in patients with aHUS in five clinical studies [6–10], in which it resolved and prevented complement-mediated TMA, improving renal function and hematologic outcomes.

Optimal duration of eculizumab therapy in aHUS has not yet been determined. The potential risk of developing TMA following discontinuation of treatment [4, 5] and the recommendation of lifelong treatment [5] have been noted in current regulatory guidance. We conducted a large prospective, long-term, observational study of patients with aHUS who were treated with eculizumab. Data from an interim analysis (cutoff date: March 28, 2015; median exposure, 45.9 months) showed a lower rate of TMA manifestations in patients who were receiving eculizumab versus patients who were not on therapy [11]. Here, we report the final results from this study.

## Methods

### Study design and patient population

**Fig. 1:**
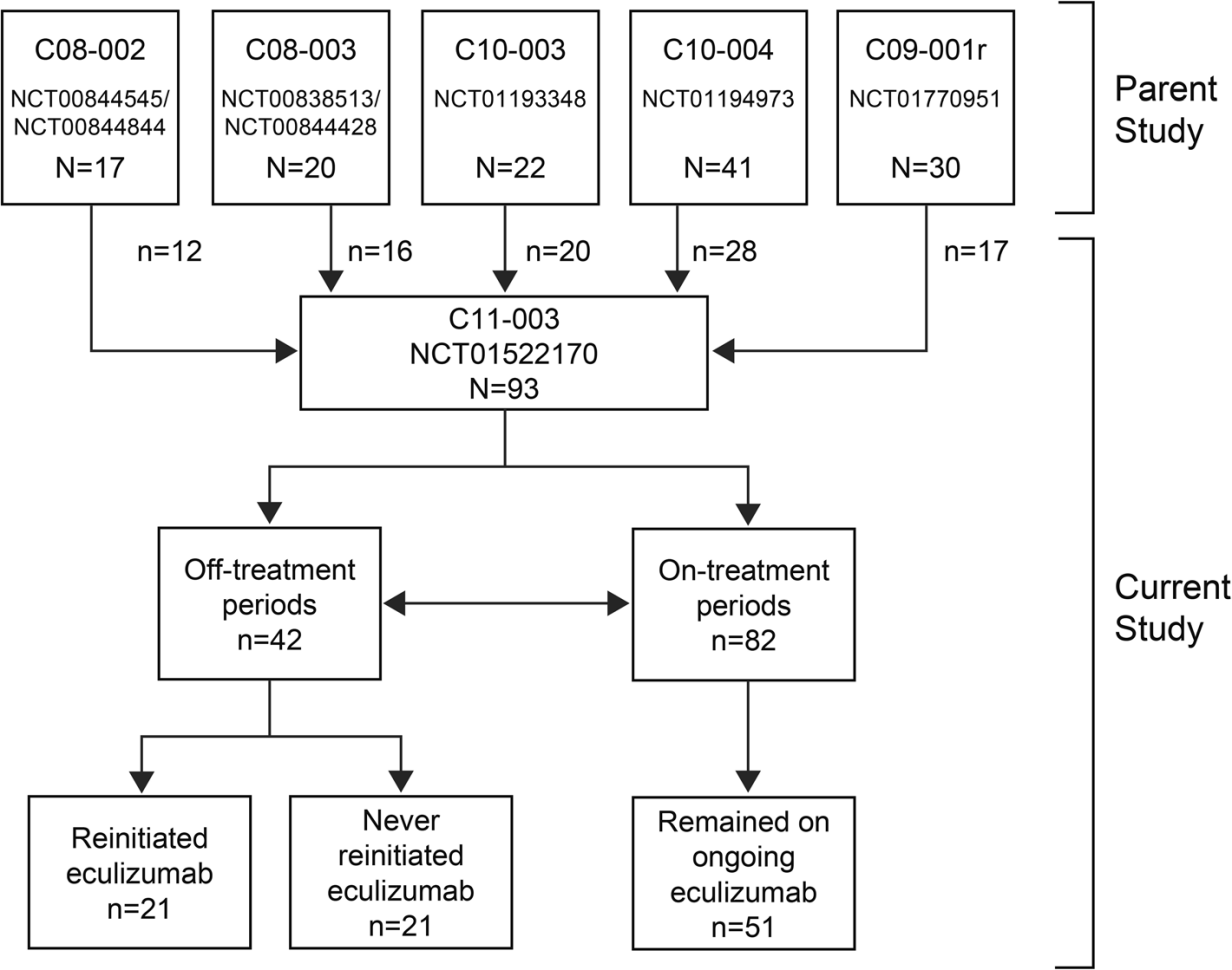
Patient disposition

This was a long-term, prospective, observational, multicenter follow-up study (NCT01522170) of patients with aHUS who were treated with at least one infusion of eculizumab in any of five previously conducted parent studies, several of which included pediatric and/or adolescent patients < 18 years of age [6–10] (Fig. 1).

Study methodology has been published previously [11], but is summarized in brief herein. Participating patients received meningococcal vaccination in the parent eculizumab trials [6, 8, 9] and were revaccinated according to their respective country guidelines. Use of antibiotic prophylaxis was not required per the study protocol but was permitted at the investigators’ discretion. Assessment of genetic and autoimmune complement abnormalities was performed in all patients upon entry into the parent studies by a centralized laboratory and was not repeated during the current study.

Baseline characteristics were recorded before the first infusion of eculizumab in the parent studies. In the parent studies, patients were administered eculizumab as per the dosing schedule described in the prescribing information. Patients were enrolled in the current study at the completion of the parent study. During the current study, changes in dosage and treatment duration were permitted at the investigators’ discretion. Clinical outcomes were compared based on eculizumab treatment status.

The first on-treatment period was defined as the date from the first infusion through 3 weeks after the last eculizumab infusion, or until patient discontinuation from the study or data cutoff (whichever occurred first). The first off-treatment period was from 3 weeks after the last infusion of eculizumab until the patient restarted eculizumab, or until patient discontinuation from the study or data cutoff (whichever occurred first). Subsequent on- and off-treatment periods were defined similarly and patient groups were not mutually exclusive.

### Study endpoints and analysis

The primary study endpoint was the rate of TMA manifestations in the current study during off and on treatment. The criteria used to define TMA are described in Table 1 [11]. Additional endpoints included change in renal function, long-term renal outcomes, and assessment of serious targeted treatment-emergent adverse events (TEAEs). Post hoc analyses were conducted to evaluate the TMA manifestation rate by comparing off- and on-treatment periods in the current study. Rates were also compared based on patient characteristics, including identified genetic or autoimmune complement abnormalities, age, history of TMA events, and transplant status.

**Table 1:** Definition of TMA manifestation (any one or more listed criteria) [11]

Type/Severity	Criteria
Laboratory values change^a^	The occurrence of a change in ≥ 2 laboratory values^b^: • Platelet count decrease ≥ 25%^c^ and < LLN • Increase in SCr ≥ 25%^c^ and > ULN • Increase in LDH ≥ 25%^c^ and > ULN
Clinical signs and symptoms of TMA^d^	Clinical signs and symptoms considered definitely related to aHUS, including: • Thrombosis • Seizure • Reduction in renal function • Proteinuria (new or worse^c^ and > 1+ or > 30 mg/dL) • Hematuria (new or worse^c^ and > 50 RBCs/HPF) • Increased hemolytic anemia • Biopsy-proven TMA • Other (eg, extrarenal TMA manifestations, including confusion, cardiovascular abnormalities, pericarditis, gastrointestinal symptoms/diarrhea)
Intervention^d^	The patient required PE/PI, dialysis, blood transfusions, or renal transplant due to a TMA manifestation

*aHUS* atypical hemolytic uremic syndrome; *HPF* high-powered field; *LDH* lactate dehydrogenase; *LLN* lower limit of normal; *PE/PI* plasma exchange/plasma infusion; *RBCs* red blood cells; *SCr* serum creatinine; *TMA* thrombotic microangiopathy; *ULN* upper limit of normal

^a^As determined by changes in laboratory parameters with ongoing follow-up

^b^Measurements were required to be confirmed by a second measurement ≥ 28 days apart with no interruption

^c^During each on period, compared with the last laboratory value during the preceding off period. During each off period, compared with the last value during the preceding on period

^d^As determined at the discretion of the investigator

Changes in renal function were measured using estimated glomerular filtration rate (eGFR). For patients who remained on eculizumab throughout the study, eGFR was compared between values determined 6 months after the initiation of eculizumab and last follow-up. For patients who discontinued eculizumab, eGFR was compared immediately prior to discontinuation and at last follow-up. For patients who discontinued but later reinitiated eculizumab, eGFR was compared immediately prior to discontinuation and at last follow-up after eculizumab reinitiation. Patients were not included in the analysis if these data were unavailable. Renal outcomes for individual patients were assessed independently by the principal investigator and two additional investigators, and reported as improved, stable, or declined; final findings were adjudicated by the principal investigator.

Serious targeted TEAEs were predefined as incidence of serious infection, meningococcal infection, sepsis, leukopenia, infusion reactions, hepatic impairment, and malignancy.

## Results

### Patient disposition

At the final data cutoff on March 30, 2017, a total of 93 patients (26 children/adolescents, 67 adults) were enrolled in the study (Fig. 1). Of these, 82 patients had on-treatment periods during the current study. Overall, 51 patients (55%; 22 children/adolescents, 29 adults) remained on eculizumab throughout the current study, while 42 (45%; 17 children/adolescents, 25 adults) had at least one off-treatment period. Twenty-one patients (50%) who discontinued eculizumab later reinitiated treatment. Reasons for reinitiation included TMA or renal impairment (*n* = 11), preparation for a kidney transplant (*n* = 5), short discontinuation period due to change in dose or missed doses (*n* = 2), administrative reasons (*n* = 2), and multiple serious AEs and a change in dosing (*n* = 1).

### Patient characteristics

Baseline patient characteristics at entry into the parent studies are presented in Table 2. Overall, 55 patients (59%) had an identified genetic or autoimmune complement abnormality. Compared with patients who discontinued eculizumab and did not reinitiate, patients who reinitiated eculizumab trended toward being younger (median 21 vs 30 years of age at first eculizumab dose), male (76% vs 52% female), treated sooner following the most recent TMA manifestation (median 0.4 vs 0.6 months), diagnosed for a longer time period before parent study entry (median 3.0 vs 0.5 months), having more prior TMA manifestations (38% vs 24% with ≥ 2 manifestations), and being more likely to have genetic or autoimmune complement abnormalities (67% vs 48%) (Table 3).

**Table 2:** Demographic and baseline clinical characteristics in the parent studies

Characteristic	Never Discontinued (*n* = 51)	Discontinued (*n* = 42)		All Patients (*N* = 93)
		Reinitiated (*n* = 21)	Not Reinitiated (*n* = 21)	
Age, at first eculizumab dose, median (range), years	23.0 (0.0, 63.0)	21.0 (0.0, 65.0)	30.0 (0.0, 80.0)	21.0 (0.0, 80.0)
Age < 12 years, n (%)	15 (29)	5 (24)	6 (29)	26 (28)
Female, n (%)	30 (59)	11 (52)	16 (76)	57 (61)
Genetic or autoimmune complement abnormality, n (%)^a^	31 (61)	14 (67)	10 (48)	55 (59)
No. of TMA manifestations prior to first eculizumab dose, n (%)				
1	30 (59)	13 (62)	16 (76)	59 (63)
≥ 2	21 (41)	8 (38)	5 (24)	34 (37)
Time from most recent TMA manifestation to the first eculizumab dose, median (range), months	1.8 (0.0, 47.4)	0.4 (0.1, 37.8)	0.6 (0.0, 19.2)	0.9 (0.0, 47.4)
Time from aHUS diagnosis to first eculizumab dose, median (range), months	18.0 (0.0, 313.3)	3.0 (0.0, 191.4)	0.5 (0.0, 178.1)	4.0 (0.0, 313.3)
No. of PE/PI sessions at latest TMA manifestation before the first eculizumab dose, median (range)	13.0 (0.0, 230.0)	7.0 (0.0, 121.0)	7.0 (0.0, 64.0)	10.5 (0.0, 230.0)
Patients with dialysis at baseline of parent study, n (%)	18 (35)	11 (52)	9 (43)	38 (41)
Patients with renal transplant prior to first eculizumab dose, n (%)	14 (28)	4 (19)	5 (24)	23 (25)

*aHUS* atypical hemolytic uremic syndrome; *CFB* complement factor B; *CFH* complement factor H; *CFI* complement factor I; *MCP* membrane cofactor protein; *PE/PI* plasma exchange/plasma infusion; *TMA* thrombotic microangiopathy

^a^Includes pathogenic variants in *C3*, *CD46* (*MCP*), *CFB*, *CFH*, and *CFI*, as well as CFH autoantibodies as determined at enrollment in parent studies

**Table 3:** Genetic and autoimmune complement abnormalities in patients in the study

Complement Abnormality by Risk Level,^a^ n (%)	Never Discontinued (*n* = 51)	Discontinued (*n* = 42)	
		Reinitiated (*n* = 21)	Not Reinitiated (*n* = 21)
High risk	25 (49)	8 (38)	6 (29)
*CFH*	14 (27)^b^	6 (29)	3 (14)^c^
*C3*	6 (12)	1 (5)	1 (5)^d^
*CFH* autoantibodies	5 (10)	1 (5)	1 (5)
*CFB*	0 (0)	0 (0)	1 (5)
Low/moderate risk	6 (12)	6 (29)	3 (14)
*CD46* (MCP)	3 (6)^e^	3 (14)	2 (10)
*CFI*	3 (6)^f^	3 (14)	1 (5)
Deletions	0 (0)	0 (0)	1 (5)
CFHR1, CHFR3	0 (0)	0 (0)	1 (5)
No identified abnormality	20 (39)	7 (33)	11 (52)

*CFB* complement factor B; *CFH* complement factor H; *CFHR1, CFHR3* complement factor H–related protein 1, complement factor H–related protein 3; *CFI* complement factor I; *MCP* membrane cofactor protein

^a^Risk stratification as proposed by Goodship et al. [17]

^b^Includes one patient who also had a *CFI* mutation, and one patient who also had a *CD46* (*MCP*) mutation

^c^Includes one patient who also had a *C3* mutation

^d^Excludes one patient who also had a *CFH* mutation

^e^Includes one patient who also had a *CFI* mutation. Excludes one patient who also had a *CFH* mutation

^f^Excludes two patients who also had *CFH* or *CD46* mutations

### Follow-up and eculizumab exposure

Duration of eculizumab therapy and follow-up time by treatment status is presented in Additional file 1: Table S1. The median (range) of follow-up overall (parent and long-term follow-up studies combined) was 65.7 (9.9, 102.2) months (> 5 years). The duration of eculizumab treatment prior to the first discontinuation was 19.6 (0.2, 86.9) months and time to reinitiation was 4.7 (0.7, 69.3) months. Patients who reinitiated eculizumab after discontinuation had an overall exposure of 56.3 (1.3, 91.3) months.

### TMA manifestations

During the study, three TMA manifestations occurred in two patients (2%) during on-treatment periods and 14 TMA manifestations occurred in 10 patients (24%) during off-treatment periods (Table 4). The rate of TMA manifestations was 1.0 per 100 patient-years during on-treatment periods and 13.5 per 100 patient-years during off-treatment periods (93% lower on treatment). All 14 TMA manifestations that occurred during off-treatment periods were reported within the first 30 months following discontinuation. In patients who discontinued eculizumab, there were no TMA manifestations during on-treatment periods, and a rate of 13.5 per 100 patient-years in off-treatment periods.

**Table 4:** TMA manifestation rates

Patient Group/Subgroup	On-Treatment Period	Off-Treatment Period	Fold Change in Rate^a^	Percent Decrease on Versus off Treatment
Overall				
Patients	*n* = 82	*n* = 42		
Patients with TMA, n (%)	2 (2)	10 (24)		
Manifestations	3	14		
Total patient-years	292.5	103.8		
Rate per 100 patient-years	1.0	13.5	13.5	93%
Patients who never discontinued				
Patients	*n* = 51	N/A		
Patients with TMA, n (%)	2 (4)	N/A		
Manifestations	3	N/A		
Total patient-years	218.2	N/A		
Rate per 100 patient-years	1.4	N/A	N/A	N/A
Patients who discontinued eculizumab				
Patients	*n* = 31	*n* = 42		
Patients with TMA, n (%)	0 (0)	10 (24)		
Manifestations	0	14		
Total patient-years	74.2	103.8		
Rate per 100 patient-years	0.0	13.5	N/A	100%
Genetic or autoimmune complement abnormality status				
Patients with complement abnormality	*n* = 51	*n* = 24		
Patients with TMA, n (%)	1 (2)	7 (29)		
Manifestations	2	9		
Total patient-years	188.3	50.1		
Rate per 100 patient-years	1.1	18.0	16.4	94%
Patients without identified complement abnormality	*n* = 31	*n* = 18		
Patients with TMA, n (%)	1 (3)	3 (17)		
Manifestations	1	5		
Total patient-years	104.1	53.7		
Rate per 100 patient-years	1.0	9.3	9.3	89%
Age at diagnosis				
Adult patients	*n* = 41	*n* = 22		
Patients with TMA, n (%)	0 (0)	3 (14)		
Manifestations	0	5		
Total patient-years	140.5	56.2		
Rate per 100 patient-years	0.0	8.9	N/A	100%
Pediatric patients^b^	*n* = 41	*n* = 20		
Patients with TMA, n (%)	2 (5)	7 (35)		
Manifestations	3	9		
Total patient-years	152.0	47.6		
Rate per 100 patient-years	2.0	18.9	9.5	89%
History of TMA events				
Single TMA	*n* = 51	*n* = 29		
Patients with TMA, n (%)	2 (4)	6 (21)		
Manifestations	3	8		
Total patient-years	192.7	70.4		
Rate per 100 patient-years	1.6	11.4	7.1	86%
Multiple TMA prior to initiation of eculizumab	*n* = 31	*n* = 13		
Patients with TMA, n (%)	1 (3)	4 (31)		
Manifestations	1	6		
Total patient-years	99.8	33.4		
Rate per 100 patient-years	1.0	18.0	18.0	94%
Transplant status				
Transplanted kidney	*n* = 21	n = 9		
Patients with TMA, n (%)	0 (0)	0 (0)		
Manifestations	0	0		
Total patient-years	76.0	24.3		
Rate per 100 patient-years	0.0	0.0	N/A	N/A
Native kidney	*n* = 61	*n* = 33		
Patients with TMA, n (%)	2 (3)	10 (30)		
Manifestations	3	14		
Total patient-years	216.5	79.5		
Rate per 100 patient-years	1.4	17.6	12.6	92%

*N/A* not applicable; *TMA* thrombotic microangiopathy

^a^During off-treatment periods compared with on-treatment periods. ^b^Defined as age < 18 years at time of diagnosis

When stratifying by patient populations (Table 4), rates of TMA during off- versus on-treatment periods were particularly high in pediatric onset patients (18.9 per 100 patient-years), patients with identified genetic or autoimmune complement abnormalities (18.0 per 100 patient-years), and patients with a history of multiple TMAs (18.0 per 100 patient-years). No TMA manifestations were reported in patients with transplanted kidneys, regardless of treatment status.

### Kidney function and long-term renal outcomes

During the first on-treatment period, eculizumab led to a rapid improvement in mean eGFR, which then remained above or near ≈60 mL/min/1.73 m^2^ during follow-up on treatment (Additional file 1: Figure S1A). In patients who remained on eculizumab treatment throughout the study and were not on chronic dialysis, median eGFR was 24.0 mL/min/1.73 m^2^ at baseline and 59.5 mL/min/1.73 m^2^ at last follow-up (Table 5). In this group of patients, dialysis was required by 18 of 51 patients (35%) at baseline and by 2 (4%) at last follow-up. Patients who discontinued had a higher mean eGFR at the time of discontinuation; over time during follow-up off eculizumab, eGFR slowly decreased but remained > 60 mL/min/1.73 m^2^ (Additional file 1: Figure S1B). In this patient subgroup, median eGFR was 12.0 mL/min/1.73 m^2^ at baseline, 92.3 mL/min/1.73 m^2^ at time of discontinuation, and 75.6 mL/min/1.73 m^2^ at last follow-up (median [range] follow-up of 31.2 [0.7, 95.1] months; Table 5). Of these patients, dialysis was required by 17 of 35 patients (48.6%) at baseline and by 5 of 35 (14.3%) at last follow-up.

**Table 5:** eGFR and dialysis over follow-up by treatment status

	Never Discontinued (*n* = 51)	Discontinued^a^ (*n* = 42)	All Patients (*N* = 93)
Patients available for eGFR analysis^b^, n	39	24	63
eGFR prior to first dose of eculizumab in parent study, mL/min/1.73 m^2^			
Mean (SD)	30.9 (26.9)	29.6 (29.1)	30.4 (27.5)
Median (range)	24.0 (8.4, 128.3)	12.0 (10.0, 105.5)	22.1 (8.4, 128.3)
eGFR at time of discontinuation^c^, mL/min/1.73 m^2^			
Mean (SD)	–	92.4 (38.6)	–
Median (range)	–	92.3 (34.2, 181.5)	–
eGFR at last follow-up, mL/min/1.73 m^2^			
Mean (SD)	65.2 (33.1)	85.9 (31.8)	73.1 (33.9)
Median (range)	59.5 (14.8, 152.2)	75.6 (40.0, 153.5)	65.7 (14.8, 153.5)
Patients on dialysis prior to first dose of eculizumab in parent study, n/N (%)	18/51 (35.3)	17/35 (48.6)	35/86 (40.7)
Patients on dialysis at last follow-up, n/N (%)	2/51 (3.9)	5/35 (14.3)	7/86 (8.1)

eGFR estimated glomerular filtration rate; SD standard deviation

^a^Patients who had eGFR values recorded in the first off-treatment period; includes patients who did and did not reinitiate eculizumab

^b^Excludes patients on chronic dialysis, defined as lasting for > 90 days without a gap of > 14 days

^c^Defined as the last eGFR value before the start of the first off-treatment period in the current study

Changes in eGFR over time were analyzed for individual patients by treatment status to assess long-term renal outcomes. When comparing long-term kidney function (Table 6), 37 patients (77%) who remained on eculizumab treatment had improved or stable renal function over time; 11 (23%) had a decline in kidney function. Overall, 14 of 35 patients (40%) who discontinued eculizumab had a decline in renal function, including 2 patients (11%) who discontinued and did not reinitiate eculizumab, and 12 patients (75%) who discontinued eculizumab and reinitiated treatment.

**Table 6:** Long-term renal outcomes by treatment status

Patients, n (%)	Never Discontinued (*n* = 48)	Discontinued, Did Not Reinitiate (*n* = 19)	Discontinued and Reinitiated (*n* = 16)	All Discontinued (*n* = 35)
Improved	17 (35)	2 (11)	0 (0)	2 (6)
Stable	20 (42)	15 (79)	4 (25)	19 (54)
Declined	11 (23)	2 (11)	12 (75)	14 (40)

Note: For patients who never discontinued eculizumab, estimated glomerular filtration rate (eGFR) was compared at 6 months post-eculizumab initiation and at last follow-up. For patients who discontinued eculizumab, eGFR was compared at the time of discontinuation and at last follow-up. For patients who reinitiated eculizumab, eGFR prior to discontinuation and at last follow-up (post-reinitiation) was compared. Three patients who remained on eculizumab and seven patients who discontinued had missing data and were excluded from this analysis. Individual patient outcomes were assessed independently by the principal investigator and two additional authors. Findings were adjudicated by the principal investigator

### Safety

Overall, eculizumab was well tolerated, and the serious targeted TEAEs are shown in Additional file 1: Table S2. Of the reported infections, cases of bacteremia, meningitis, meningococcal infection, pneumococcal infection, mycoplasma pneumonia, and sepsis were determined to be related to eculizumab treatment by the investigators. During the current and parent studies, patients reported three definite and one possible meningococcal infections, but all continued eculizumab and recovered from the infection (Table 7). There were three deaths in the current and parent studies; none were considered related to eculizumab (Table 7)

**Table 7:** Safety events from first dose of eculizumab in parent study

Outcome	Sex	Age Category	Description
Experienced meningococcal infection while on eculizumab treatment	Female	20–29	• *CFH* mutation • Vaccinated with Mencevax® (ACYW135), not on prophylactic antibiotics • Meningococcal infection serogroup B identified • Received antibiotics for treatment of infection, which resolved after 9 days, and continued on eculizumab
	Female	20–29	• *CFH* mutation, renal transplant • Vaccinated with Mencevax® (ACYW135), not on prophylactic antibiotics • Meningococcal infection serogroup W135 identified • Received antibiotics for treatment of infection, which resolved after 17 days, and continued on eculizumab
	Male	20–29	• No identified complement abnormality at diagnosis; renal transplant • Vaccinated with Menveo® (ACYW135), prophylactic antibiotics • Meningococcal infection serogroup B identified • Received antibiotics for treatment of infection, which resolved after 10 days, and continued on eculizumab
	Male	13–19	• *C3* mutation, renal transplant • Vaccinated with Menactra®, prophylactic antibiotics • Clinical presentation was consistent with possible meningococcal infection (sore throat, knee pain and swelling, skin lesions), but all blood cultures were negative • Received antibiotics for treatment of infection, which resolved after 6 days, and continued on eculizumab
Death^a^	Male	30–39	• *C3* mutation, renal transplant, hemorrhagic gastric ulcer s/p gastrotomy • Had discontinued eculizumab approximately 6 months prior to death • *Cause of death:* Severe intensive care complications and multiorgan dysfunction secondary to gastrointestinal hemorrhage, lithiasic cholecystitis, and sepsis
	Male	< 5	• No complement abnormality identified at diagnosis; renal failure, respiratory distress, hepatitis, and seizure disorder • Patient^b^ experienced abdominal pain, series of infections and bacterial infection after 10 months on eculizumab at a reduced dose; had seizures attributed to metabolic encephalopathy • *Cause of death:* Hypoxia due to diffuse alveolar hemorrhage
	Female	< 5	• No complement abnormality identified at diagnosis; renal and cardiac failure, pulmonary hypertension, cardiomyopathy • Was on dialysis at diagnosis and was treated with eculizumab for 2 months but discontinued due to “lack of efficacy” • Patient experienced a TMA manifestation with multiorgan failure • *Cause of death:* Respiratory failure led to cardiac arrest and anoxic brain injury after being off treatment for 7 months

*aHUS* Atypical hemolytic uremic syndrome, *CFH* Complement factor H, *TMA* Thrombotic microangiopathy

^a^No death was considered related to eculizumab

^b^Patient also described in Additional file 1: Table S2

.

## Discussion

We have conducted the largest and longest prospective cohort study of eculizumab in aHUS patients, across all age groups. Data from an earlier, interim analysis (cutoff date: March 28, 2015, median exposure, 45.9 months) [11] provided preliminary information on rates of TMA manifestations occurring both off and on eculizumab treatment. Here, we present the final data from this study, providing 2 additional years of follow-up, subgroup analysis that may inform risk profiles, and evaluation of the long-term renal consequences.

In the previous interim analysis [11], TMA manifestations were defined as either clinical signs and symptoms of TMA, interventions, or changes in laboratory values. Although there is no standard definition of TMA, which may be associated with varying degrees of clinical deterioration and outcomes, it is unlikely that changes in single laboratory values are considered to be TMA manifestations by treating clinicians outside of this trial. Thus, in this final analysis, we employed the more stringent definition (ie, excluding TMA defined by changes in a single laboratory criterion) used in the previous interim report [11]. Based on this definition, the rates in this study were 13.5-fold higher during off-treatment periods compared with on-treatment periods, consistent with the interim report.

Ability to identify patients at particular risk for TMA while off treatment would be beneficial for clinicians when deciding on long-term management, aiming to reduce risk of TMA relapse. Additional patient enrollment and length of follow-up allowed for evaluation of TMA manifestation rates by patient subgroups, although the study was not specifically designed to facilitate these comparisons. TMA manifestation rates were particularly high off treatment in patients with identified complement genetic or autoimmune abnormalities. These findings are consistent with previous long-term retrospective analyses of the natural history of aHUS in the pre-eculizumab era [3, 12] as well as previous prospective and retrospective analyses of eculizumab discontinuation [13–16]. Our current findings also highlight new patient subgroups that may be at particular risk for TMA relapse when taken off treatment, including patients with pediatric onset, and/or those with a history of multiple TMAs. Due to the historically higher mortality rate in pediatric patients and patients diagnosed before adulthood, it is crucial to protect these patients from additional TMA manifestations [3]. When comparing findings from the current analysis to those from a retrospective study of eculizumab discontinuation in a French cohort [15], previous history of multiple TMA manifestations has been consistently identified as a factor of potential importance for TMA risk while off treatment.

Patients with kidney transplants were excluded from the analysis by Fakhouri et al. [15], but TMA events after discontinuation of eculizumab among patients with aHUS following kidney transplants have been reported elsewhere [16]. Interestingly, in this study, no TMA manifestations were reported off or on treatment in patients who had transplants. When evaluating profiles of patients with transplants, patients who remained on therapy were more likely to have higher-risk complement abnormalities (ie, *CFH*, *CFB*, and *C3* mutations) and/or multiple history of graft loss. This could suggest that, in this study, transplant patients who discontinued had a lower risk profile overall. Recent guidance from Kidney Disease: Improving Global Outcomes (KDIGO) [17] includes the recommendation that transplant patients, and particularly those with history of previous graft loss, should not discontinue eculizumab because of historically high risk of recurrence and graft loss in the pre-eculizumab era [18]. Data from a case series by Levi et al. [19] document risk of TMA post-eculizumab discontinuation in a transplant patient. However, Duineveld et al. [20] suggest that transplantation in aHUS is possible without prophylactic eculizumab, particularly with living donors, use of lower-dose immunosuppressive regimens, and strict blood pressure control to mitigate endothelial injury. Data from the Global aHUS Registry suggests better outcomes in patients with transplants who initiated eculizumab prior to transplant [21]. Further studies are required for this patient subgroup.

With respect to consequences of TMA and long-term renal outcomes, this study includes the longest-term follow-up (median, 65.7 months of follow up [ie, > 5 years]) of changes in eGFR ever reported in patients with aHUS. Renal function loss following TMA manifestation was improved with eculizumab, and this improvement was maintained for a median of 74 months (> 6 years) of follow-up. At the time of discontinuation, patients who discontinued eculizumab had higher eGFRs than the on-treatment plateau value in patients who never discontinued. Because discontinuation was at the investigators’ discretion, we can only speculate as to why this was the case. It may reflect decreased concern regarding risk of TMA in patients with fairly normal kidney function, and a choice was made to discontinue treatment.

We observed a trend toward a decline in kidney function in the group of patients who discontinued eculizumab. This was observed when evaluating the median eGFR over time and, more importantly, when evaluating individual patients during the period when eculizumab was discontinued. Patients who discontinued eculizumab were almost twice as likely to have a decline in kidney function than patients who never discontinued (40% vs 23%) and were less likely to have an improvement in kidney function (6% vs 35%). Because this decline was irrespective of whether a patient had a reported TMA event, it is possible that some patients had “subclinical” TMA activity after eculizumab discontinuation. An analysis of individual patient outcomes in the pediatric patients in this study (Unpublished data, Pape et al., European Society for Paediatric Nephrology, 2018) revealed that after eculizumab discontinuation, some patients maintained renal function but some returned to pre-eculizumab eGFR values, particularly following a TMA event. Perhaps not surprisingly, those patients who reinitiated eculizumab were especially likely to have a decline in kidney function when not receiving eculizumab.

Half of the patients who discontinued eculizumab later reinitiated during the study, mostly due to TMA/renal impairment, or in preparation for a kidney transplant. One patient had multiple serious adverse events and a change in dosing; treatment was reinitiated to salvage his condition, but the patient eventually died during this study. The two patients categorized as “due to change in dose or missed doses” likely would not have been categorized as having discontinued in clinical practice, but were retained in this category based on the definition in the statistical analysis plan of this study. Shifting these two patients to the group of patients who remained on eculizumab based on sensitivity analysis would not have changed outcomes, because no TMA manifestations were reported in these patients.

There were differences between the subgroups of patients who reinitiated eculizumab and those who discontinued eculizumab and never reinitiated therapy. Interestingly, a lower proportion of females reinitiated treatment (52% of the subgroup) than those who never reinitiated treatment (76% of the subgroup). Patients who reinitiated treatment were younger and were more likely to have identified complement genetic or autoimmune abnormalities and a history of multiple TMA manifestations, which is similar to the subgroup of patients who never discontinued. Evidence from a pooled, post hoc analysis of the eculizumab clinical trial program in aHUS [22] demonstrated that younger age is among the factors associated with better outcomes on eculizumab. In that analysis, earlier initiation of eculizumab led to improved renal recovery [22]. It is yet to be determined how TMA history before initiation of therapy may affect the disease course after discontinuation.

The safety profile was consistent with those reported in the parent trials [6–9]. Three deaths were reported and were determined to be unrelated to eculizumab treatment. Three definite and one possible meningococcal infections related to eculizumab were reported during this study, and resolved with appropriate antibiotic treatment. The number of meningococcal infections may be related to the sample size in the study, the severity of the patient population included in the trial population [6–9], as well as that the events largely occurred before the more widespread use of antibiotic prophylaxis. A larger post-marketing analysis of safety events and, specifically, meningococcal infections has been performed in all patients treated with eculizumab for paroxysmal nocturnal hemoglobinuria and aHUS worldwide. This report provided 10 years of safety information and included a meningococcal infection rate of 0.25 per 100 patient-years, which tended to decrease in frequency over that time period [23]. Clinicians should continue to consult regulatory guidance [4, 5] regarding patient counseling of the eculizumab benefit/risk profile, early signs of meningococcal disease, and processes for seeking immediate medical care. The Centers for Disease Control and Prevention has noted that antibiotic prophylaxis is generally considered to be safe, and suggests that clinicians could consider its use for the duration of eculizumab treatment [24].

One limitation of this study is that discontinuation of eculizumab was not done randomly but at the discretion of investigators and patients, and other possible management strategies of aHUS were not evaluated. Hence, the results must be interpreted cautiously. However, it seems likely that patients who were perceived to be at higher risk of TMA would be less likely to discontinue therapy. Changes in dosage were permitted at the investigators’ discretion and also were not randomized. Another limitation is that small patient numbers and TMA manifestations prevented robust analysis of patient subgroups. In addition, requirement of serum creatinine level to be both increased by ≥ 25% and above the upper limit of normal to qualify as a TMA manifestation may have resulted in missed TMA among patients with low muscle mass and low baseline creatinine levels. Finally, identification and validation of sensitive biomarkers of early signs of TMA recurrence may assist in managing these patients long-term for optimal outcomes.

## Conclusions

In summary, this long-term, prospective study confirms the efficacy and safety of eculizumab in the treatment of aHUS, especially as it relates to improvement and maintenance of stable kidney function over 6 years and a very low rate of TMA during that time. Discontinuation of eculizumab was associated with higher risk of TMA and trends toward decreases in renal function over time, despite very high mean eGFR at the time of discontinuation. These findings confirm a similar analysis recently conducted in the patient population enrolled in the Global aHUS Registry [25]. Patient subgroups at the highest potential risk for TMA post-discontinuation were identified and included pediatric disease onset, identified genetic or autoimmune complement abnormalities, and a history of multiple TMAs. Consideration of such risk factors is important during treatment decision-making for patients with aHUS, and close monitoring for signs of TMA and rapid reinitiation of treatment at early signs of TMA is needed for patients who discontinue eculizumab therapy. Under these conditions, discontinuation of eculizumab with careful monitoring may be an option for select patients with aHUS.

## Additional file

Additional file 1: **Table S1.** Follow-up by treatment status. **Table S2.** Serious targeted TEAEs during current study. **Figure S1A** and **B.** Effects on renal function based on eGFR.

## Abbreviations

aHUS: Atypical hemolytic uremic syndrome
CFB: Complement factor B
CFH: Complement factor H
CFHR1, CFHR3: Complement factor H–related protein 1, complement factor H–related protein 3
CFI: Complement factor I
eGFR: Estimated glomerular filtration rate
HPF: High-powered field
KDIGO: Kidney Disease: Improving Global Outcomes
LDH: Lactate dehydrogenase
LLN: Lower limit of normal
N/A: Not applicable
PE/PI: Plasma exchange/plasma infusion
RBC: Red blood cell
SCr: Serum creatinine
SD: Standard deviation
TEAE: Treatment-emergent adverse event
TMA: Thrombotic microangiopathy
ULN: Upper limit of normal

## References

[1] Fakhouri F, Zuber J, Fremeaux-Bacchi V, Loirat C (2017). Haemolytic uraemic syndrome. Lancet..

[2] Campistol JM, Arias M, Ariceta G, Blasco M, Espinosa L, Espinosa M, Grinyo JM, Macia M, Mendizabal S, Praga M, Roman E, Torra R, Valdes F, Vilalta R, Rodriguez de Cordoba S (2015). An update for atypical haemolytic uraemic syndrome: diagnosis and treatment. A consensus document. Nefrologia..

[3] Fremeaux-Bacchi V, Fakhouri F, Garnier A, Bienaime F, Dragon-Durey MA, Ngo S, Moulin B, Servais A, Provot F, Rostaing L, Burtey S, Niaudet P, Deschenes G, Lebranchu Y, Zuber J, Loirat C (2013). Genetics and outcome of atypical hemolytic uremic syndrome: a nationwide French series comparing children and adults. Clin J Am Soc Nephrol.

[4] US Food and Drug Administration (2018). Soliris (eculizumab) [prescribing information].

[5] European Medicines Agency (2017). Soliris (eculizumab) [summary of product characteristics].

[6] Legendre CM, Licht C, Muus P, Greenbaum LA, Babu S, Bedrosian C, Bingham C, Cohen DJ, Delmas Y, Douglas K, Eitner F, Feldkamp T, Fouque D, Furman RR, Gaber O, Herthelius M, Hourmant M, Karpman D, Lebranchu Y, Mariat C, Menne J, Moulin B, Nurnberger J, Ogawa M, Remuzzi G, Richard T, Sberro-Soussan R, Severino B, Sheerin NS, Trivelli A, Zimmerhackl LB, Goodship T, Loirat C (2013). Terminal complement inhibitor eculizumab in atypical hemolytic-uremic syndrome. N Engl J Med.

[7] Licht C, Greenbaum LA, Muus P, Babu S, Bedrosian CL, Cohen DJ, Delmas Y, Douglas K, Furman RR, Gaber OA, Goodship T, Herthelius M, Hourmant M, Legendre CM, Remuzzi G, Sheerin N, Trivelli A, Loirat C (2015). Efficacy and safety of eculizumab in atypical hemolytic uremic syndrome from 2-year extensions of phase 2 studies. Kidney Int.

[8] Greenbaum Larry A., Fila Marc, Ardissino Gianluigi, Al-Akash Samhar I., Evans Jonathan, Henning Paul, Lieberman Kenneth V., Maringhini Silvio, Pape Lars, Rees Lesley, van de Kar Nicole C.A.J., Vande Walle Johan, Ogawa Masayo, Bedrosian Camille L., Licht Christoph (2016). Eculizumab is a safe and effective treatment in pediatric patients with atypical hemolytic uremic syndrome. Kidney International.

[9] Fakhouri F, Hourmant M, Campistol JM, Cataland SR, Espinosa M, Gaber AO, Menne J, Minetti EE, Provot F, Rondeau E, Ruggenenti P, Weekers LE, Ogawa M, Bedrosian CL, Legendre CM (2016). Terminal complement inhibitor eculizumab in adult patients with atypical hemolytic uremic syndrome: a single-arm, open-label trial. Am J Kidney Dis.

[10] Vilalta R, Al-Akash S, Davin J, Diaz J, Gruppo R, Hernandez J, Jungraithmayr T, Langman C, Lapeyraque A, Macher M, Rodig N, Sherbotie J, Sherwinter J, Simonetti G, Smith J, Thornburg C, Wuhl E (2012). Eculizumab therapy for pediatric patients with atypical hemolytic uremic syndrome: efficacy and safety outcomes of a retrospective study [abstract 1155]. Haematologica..

[11] Menne J, Delmas Y, Fakhouri F, Kincaid JF, Licht C, Minetti EE, Mix C, Provot F, Rondeau E, Sheerin NS, Wang J, Weekers LE, Greenbaum LA. Eculizumab prevents thrombotic microangiopathy in patients with atypical haemolytic uraemic syndrome in a long-term observational study. Clin Kidney J. 2018. [Epub ahead of print]. 10.1093/ckj/sfy035 PMC6452204 30976396

[12] Noris M, Caprioli J, Bresin E, Mossali C, Pianetti G, Gamba S, Daina E, Fenili C, Castelletti F, Sorosina A, Piras R, Donadelli R, Maranta R, van der Meer I, Conway EM, Zipfel PF, Goodship TH, Remuzzi G (2010). Relative role of genetic complement abnormalities in sporadic and familial aHUS and their impact on clinical phenotype. Clin J Am Soc Nephrol.

[13] Ardissino G, Possenti I, Tel F, Testa S, Salardi S, Ladisa V (2015). Discontinuation of eculizumab treatment in atypical hemolytic uremic syndrome: an update. Am J Kidney Dis.

[14] Ardissino G, Testa S, Possenti I, Tel F, Paglialonga F, Salardi S, Tedeschi S, Belingheri M, Cugno M (2014). Discontinuation of eculizumab maintenance treatment for atypical hemolytic uremic syndrome: a report of 10 cases. Am J Kidney Dis.

[15] Fakhouri F, Fila M, Provot F, Delmas Y, Barbet C, Chatelet V, Rafat C, Cailliez M, Hogan J, Servais A, Karras A, Makdassi R, Louillet F, Coindre JP, Rondeau E, Loirat C, Fremeaux-Bacchi V (2017). Pathogenic variants in complement genes and risk of atypical hemolytic uremic syndrome relapse after eculizumab discontinuation. Clin J Am Soc Nephrol.

[16] Wijnsma Kioa L, Duineveld Caroline, Volokhina Elena B, van den Heuvel Lambertus P, van de Kar Nicole C A J, Wetzels Jack F M (2017). Safety and effectiveness of restrictive eculizumab treatment in atypical haemolytic uremic syndrome. Nephrology Dialysis Transplantation.

[17] Goodship TH, Cook HT, Fakhouri F, Fervenza FC, Fremeaux-Bacchi V, Kavanagh D, Nester CM, Noris M, Pickering MC, Rodriguez de Cordoba S, Roumenina LT, Sethi S, Smith RJ (2017). Atypical hemolytic uremic syndrome and C3 glomerulopathy: conclusions from a "Kidney Disease: Improving Global Outcomes" (KDIGO) Controversies Conference. Kidney Int.

[18] Le Quintrec M, Zuber J, Moulin B, Kamar N, Jablonski M, Lionet A, Chatelet V, Mousson C, Mourad G, Bridoux F, Cassuto E, Loirat C, Rondeau E, Delahousse M, Fremeaux-Bacchi V (2013). Complement genes strongly predict recurrence and graft outcome in adult renal transplant recipients with atypical hemolytic and uremic syndrome. Am J Transplant.

[19] Levi C, Fremeaux-Bacchi V, Zuber J, Rabant M, Devriese M, Snanoudj R, Scemla A, Amrouche L, Mejean A, Legendre C, Sberro-Soussan R (2017). Midterm outcomes of 12 renal transplant recipients treated with eculizumab to prevent atypical hemolytic syndrome recurrence. Transplantation..

[20] Duineveld C, Verhave JC, Berger SP, van de Kar N, Wetzels JFM (2017). Living donor kidney transplantation in atypical hemolytic uremic syndrome: a case series. Am J Kidney Dis.

[21] Siedlecki Andrew M., Isbel Nicole, Vande Walle Johan, James Eggleston Jennifer, Cohen David J., Licht Christoph, Frémeaux-Bacchi Véronique, Ariceta Gema, Ardissino Gianluigi, Fakhouri Fadi, Greenbaum Larry, Johnson Sally, Schaefer Franz, Scully Marie Ann, Woodward Leonard, Ogawa Masayo, Gasteyger Christoph, Blasco Miquel, Cresseri Donata, Generolova Galina, Webb Nicholas, Hirt-Minkowski Patricia, Kozlovskaya Natalya Lvovna, Landau Danny, Lapeyraque Anne-Laure, Loirat Chantal, Mache Christoph, Malina Michal, Martola Leena, Massart Annick, Rondeau Eric, Sartz Lisa (2019). Eculizumab Use for Kidney Transplantation in Patients With a Diagnosis of Atypical Hemolytic Uremic Syndrome. Kidney International Reports.

[22] Vande Walle J, Delmas Y, Ardissino G, Wang J, Kincaid JF, Haller H (2017). Improved renal recovery in patients with atypical hemolytic uremic syndrome following rapid initiation of eculizumab treatment. J Nephrol.

[23] Socie G, Caby-Tosi MP, Marantz JL, Cole JA, Bedrosian CL, Gasteyger C, Mujeebuddin A, Hillmen P, Vande Walle J, Haller H. Eculizumab in paroxysmal nocturnal haemoglobinuria and atypical haemolytic uraemic syndrome: 10-year pharmacovigilance analysis. Br J Haematol. 2019. [Epub ahead of print]. 10.1111/bjh.15790 PMC6594003 30768680

[24] McNamara LA, Topaz N, Wang X, Hariri S, Fox L, MacNeil JR (2017). High risk for invasive meningococcal disease among patients receiving eculizumab (soliris) despite receipt of meningococcal vaccine. MMWR Morb Mortal Wkly Rep.

[25] Ariceta G, Ardissino G, Sartz L, Fakhouri F, Gasteyger C, Al-Dakkak I (2018). Thrombotic microangiopathy frequency in patients with atypical HUS: discontinuing vs remaining on eculizumab treatment [abstract FR-OR017]. J Am Soc Nephrol.

