# Supplementary material for: Outcomes in patients with atypical hemolytic uremic syndrome treated with eculizumab in a long-term observational study
Source: BMC Nephrol. 2019 Apr 10;20:125. doi: 10.1186/s12882-019-1314-1 (PMC6456946; doi:10.1186/s12882-019-1314-1)

**Table S1.** Follow-up by treatment status

|  | **Never Discontinued**  **(n=51)** | **Discontinued (n=42)** | | **All Patients**  **(N=93)** |
| --- | --- | --- | --- | --- |
|  |  | **Reinitiated**  **(n=21)** | **Not Reinitiated**  **(n=21)** |  |
| **Total duration of follow-up, median (range) months** | 74.4 (9.9, 94.1) | 65.4 (31.2, 96.4) | 63.0 (12.1, 102.2) | 65.7 (9.9, 102.2) |

**Table S2.** Serious targeted TEAEs during current study^a^

|  | **On Treatment**  **(n=82)** | **Off Treatment**  **(n=42)** |
| --- | --- | --- |
| **Total patient-years** | 292.5 | 103.8 |
| **Patients with ≥1 event, n (%)** | 24 (29.3) | 19 (45.2) |
| **Serious infections, nonencapsulated bacteria or fungi** |  |  |
| Acute sinusitis | 1 (0.3) | 0 (0) |
| *Aspergillus* infection^b^ | 1 (0.3) | 0 (0) |
| Enterococcal infection^b^ | 1 (0.3) | 0 (0) |
| *Escherichia coli* infection^b^ | 1 (0.3) | 0 (0) |
| *Candida* infection | 0 (0) | 1 (1.0) |
| Bacterial infection^c^ | 1 (0.3) | 0 (0) |
| **Pyelonephritis and urinary tract infections^d^** | 12 (4.1) | 0 (0) |
| **Sepsis^e^** | 6 (2.1) | 4 (3.9) |
| **Lung infection^f^** | 4 (1.4) | 1 (1.0) |
| **Viral infections, nonspecific, or upper respiratory infections^g^** | 4 (1.4) | 4 (3.9) |
| **Gastroenteritis, bacterial** | 1 (0.3) | 0 (0) |
| **Gastroenteritis, viral** | 1 (0.3) | 0 (0) |
| **Viral meningitidis** | 1 (0.3) | 0 (0) |
| **Leukopenia** | 1 (0.3) | 0 (0) |
| **Malignant neoplasm** | 0 (0) | 1 (1.0) |
| **Staphylococcal infection** | 0 (0) | 1 (1.0) |
| **Hepatic impairment** | 0 (0) | 0 (0) |

*TEAEs* treatment-emergent adverse events.

Note: Serious targeted TEAEs are reported as n (rate per 100 patient-years).

^a^The meningococcal infections and deaths are described separately in Table 7

^b^The *Aspergillus* infection, enterococcal infection, and *E. coli* infection occurred in the same patient during the same time period as a peritonitis infection.

^c^Multiple infections occurring consecutively including peritonitis, two viral upper respiratory infections, sepsis, urinary tract infection, and a final fatal unspecific bacterial infection. Patient is also described in Table 7.

^d^Includes one microsporidia infection.

^e^Includes *Enterobacter* bacteremia, urosepsis, and pneumococcal infection in central lines.

^f^Includes pneumonia, mycoplasmal pneumonia, by unspecified organism, and unspecified lung infection.

^g^Includes one otitis media.

**Fig. S1.** Effects on renal function based on eGFR. (**A**) First on-treatment period. Results displayed for n>5. Excludes patients on chronic dialysis. Baseline (study week 0) was defined as the first infusion of eculizumab in the parent study. eGFR values were imputed as 10 mL/min/1.73 m^2^ while a patient was on dialysis. All eGFR values after post-dose kidney transplant were excluded. Patient values were censored at time of eculizumab discontinuation. (**B**) First off-treatment period. Results displayed for n>5. Excludes patients on chronic dialysis. Baseline (study week 0) was defined as the last value collected during the first on-treatment period. eGFR values were imputed as 10 mL/min/1.73 m^2^ while a patient was on dialysis. All eGFR values after post-dose kidney transplant were excluded. Patient values were censored at time of eculizumab reinitiation. eGFR, estimated glomerular filtration rate; SD, standard deviation.


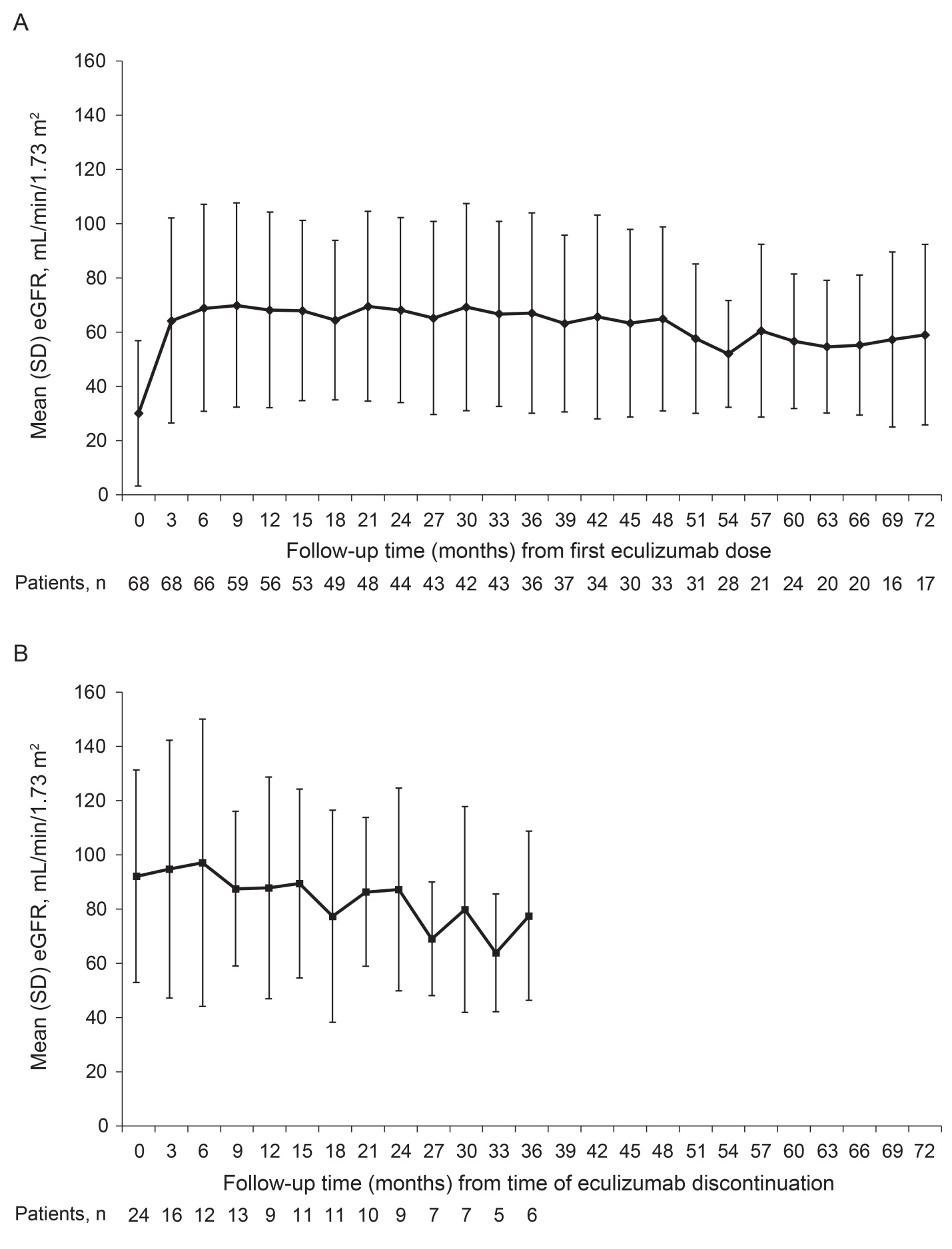

Supplement: Supplementary file 1 — Table S1. Follow-up by treatment status. Table S2. Serious targeted TEAEs during current study. Figure S1A and B. Effects on renal function based on eGFR. [file 12882_2019_1314_MOESM1_ESM.docx]
